# Evaluating the effects of the free healthcare policy on clinical visits and malaria among children in Burkina Faso: a modeling study of past trends and future forecasts

**DOI:** 10.1186/s41256-025-00455-5

**Published:** 2025-12-15

**Authors:** André Lin Ouédraogo, Marie-Jeanne Offosse, Julie Zhang, Sylvain Toé, Pierre Yaméogo

**Affiliations:** 1 Baobab Shift, Seattle, WA USA; 2 Former Institute for Disease Modeling, Gates Foundation, Seattle, WA USA; 3 Former Ministry of Health and Public Hygiene, Ouagadougou, Burkina Faso; 4 Country Office, ThinkWell Institute, Ouagadougou, Burkina Faso; 5 Department of Statistics, Stanford University, San Francisco, USA; 6 Terres Des Hommes, Ouagadougou 01, 01 BP 2212, Ouagadougou, Burkina Faso; 7 Former Technical Secretary for Health Financing Reforms, Ministry of Health and Public Hygiene, Ouagadougou, Burkina Faso; 8 Health Financing Advisor, Ministry of Health, Djibouti, Djibouti

**Keywords:** Burkina Faso, Free healthcare policy, Primary healthcare, Children under five, Malaria, Equity, Difference-in-differences, Bayesian modeling, Gratuité, Distance

## Abstract

**Background:**

Access to primary healthcare remains a major challenge in sub-Saharan Africa. In 2016, Burkina Faso introduced the free healthcare policy, eliminating financial barriers by offering free healthcare services for children under five and pregnant women. While previous studies have reported increased healthcare utilization under the policy, its effects on health outcomes remains unassessed. Here, we evaluate the free healthcare policy’s effects on three key indicators: frequency of clinical visits, malaria prevalence, and severe malaria.

**Methods:**

Routine health and demographic data for children under five were collected from health facilities using the government’s Electronic Medical Record (EMR) platform. Generalized Linear Mixed Effects Models (GLMM) with a Differences-in-Differences design assessed changes in the three indicators between baseline (2015) and 2018 while Bayesian hierarchical models were used to forecast trends through 2020.

**Results:**

Analysis of 344,935 clinical visits from 199,664 children across 192 villages showed an increase in healthcare utilization, with clinical visits rising from 1.1 to 2 per child between 2015 and 2018 (*p* < 0.001) resulting in a percentage increase of 82%. However, disparities persisted, as children living within 5 km of a health facility accessed more care than those in remote areas (2.2 vs. 1.7 visits, *p* < 0.001). Malaria prevalence decreased from 98 to 80% (*p* < 0.001), and severe malaria declined from 6% to 2.2% (*p* < 0.001) during the same period. Bayesian forecasts indicated continued increases in clinical visits (from 2.36 in 2019 to 2.75 in 2020) and further declines in malaria prevalence (to 64%) and severe malaria (to 1.02%) by 2020.

**Conclusions:**

Our study highlights the transformative effects of the free healthcare policy in improving healthcare access and outcomes for children under five in Burkina Faso. However, inequities in access remain a challenge. Strengthening community health worker programs and expanding community health activities are critical to addressing these gaps. The findings offer valuable insights for policymakers in Burkina Faso and contribute to global efforts to leverage health reforms to improve malaria control and progress toward universal health coverage.

**Supplementary Information:**

The online version contains supplementary material available at 10.1186/s41256-025-00455-5.

## Introduction

Access to equitable healthcare remains a persistent challenge in Burkina Faso [1, 2], particularly for vulnerable groups such as children under five and pregnant women, reflecting broader trends across sub-Saharan Africa [3–5]. The ratio of health infrastructures in the country was estimated at 1.3 per 10,000 inhabitants in 2014 [1], with approximately 42% of the population living more than 4 km from the nearest facility in 2015 [6]. Despite these challenges, Burkina Faso has made significant strides in strengthening its health system and improving health outcomes through targeted interventions. Notably, the country adopted the Expanded Program on Immunization (EPI) in the 1980s [7], in line with World Health Organization (WHO) recommendations [8], introducing key vaccines such as the oral polio vaccine (OPV) and the measles vaccine to combat preventable diseases [9–11]. In the 2000s, additional measures were introduced to address endemic health challenges. These included the rollout of Insecticide-Treated Nets (ITNs) to reduce malaria transmission and mortality [12, 13] and the launch of Seasonal Malaria Chemoprevention (SMC) in 2014, which involved administering antimalarial drugs to children under five during high transmission seasons to prevent severe cases [14]. To further improve access to essential health services, Burkina Faso expanded its Community Health Worker (CHW) program in 2016, emphasizing preventive care and health education at the community level [15–18].

While a partial subsidy covering 80% of obstetrical delivery costs existed between 2006 and 2015 in health centers and referral hospitals for pregnant women [19], routine outpatient services for under-five children and pregnant women still required user payments before 2016. The introduction of the free healthcare policy, locally known as the Gratuité policy, in 2016, marked a transformative step in Burkina Faso’s health reforms. For consistency and cultural relevance, we refer to it throughout this paper by its local name [20]. Supported by an annual investment of $65 million [21, 22], this policy sought to eliminate financial barriers by providing free consultations, diagnostic tests, medications, and treatments for pregnant women and children under five [23]. The policy was particularly critical in addressing major health challenges such as malaria, diarrhea, and pneumonia among children under five [24–27], with objectives centered on improving healthcare utilization, ensuring timely treatment, reducing disease burden, and achieving sustainable health outcomes. While studies evaluating the Gratuité policy have documented increases in healthcare utilization among mothers and children, the effects of the policy on health outcomes, particularly in reducing disease prevalence and severe cases has not been assessed [22, 28–30].

This study aims to fill this gap by evaluating the effects of the Gratuité policy’s reform on healthcare access and health outcomes for children under five in Burkina Faso. Specifically, it focuses on three key indicators: the frequency of clinical visits, the prevalence of malaria diagnosed through Rapid Diagnostic Tests (RDTs), and the prevalence of severe malaria cases. Using data from the Gratuité policy’s baseline year (2015) to 2018, this study examines changes in healthcare-seeking behavior and disease outcomes, particularly in remote areas where access remains a challenge. The findings will inform evidence-based policymaking in Burkina Faso and enrich global dialogue on how health reforms can advance health equity and improve outcomes in under-resourced health systems.

## Methods

### Setting and study design

**Fig. 1:**
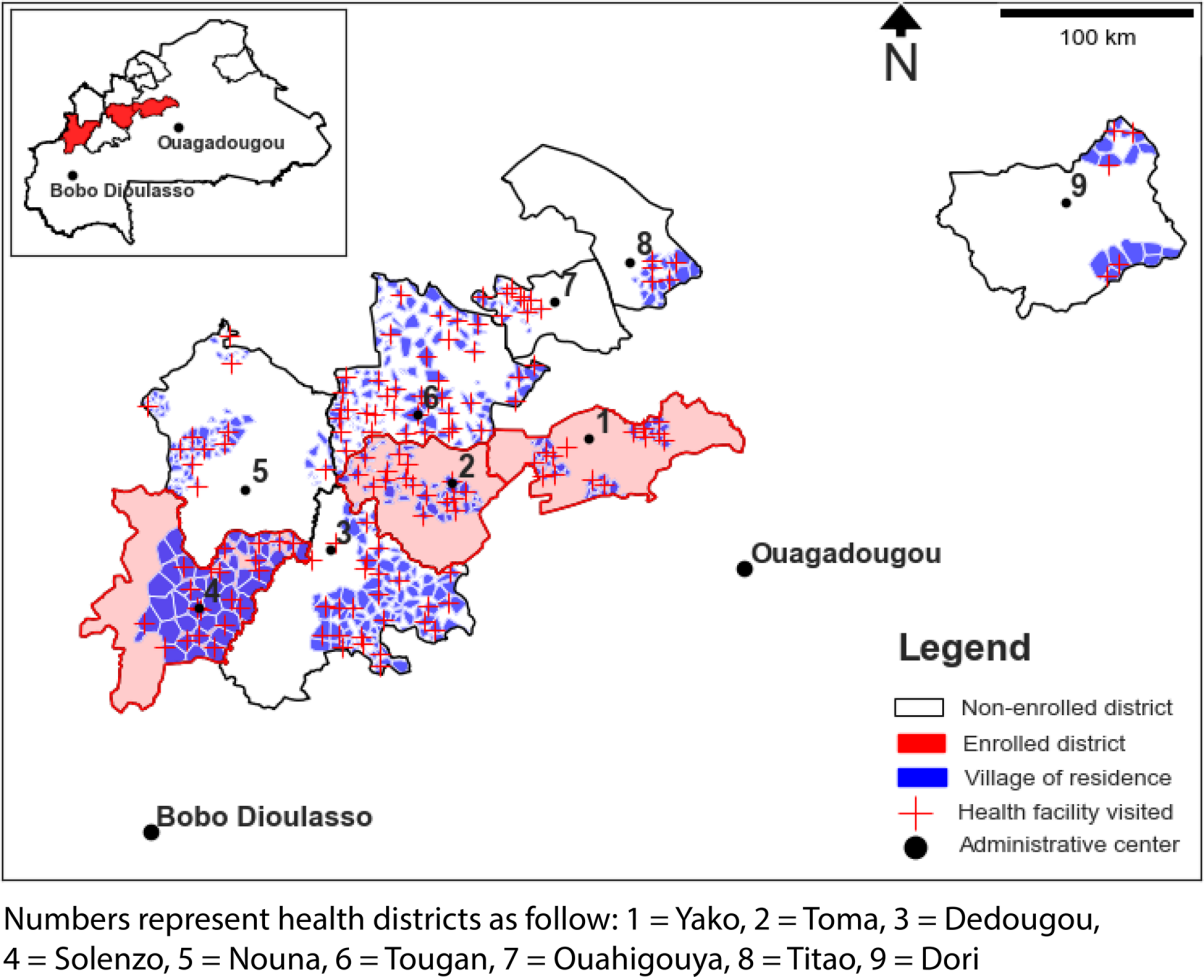
Setting

The study focuses on children under five residing in the health districts of Dori, Dedougou, Nouna, Ouahigouya, Titao, Toma, Tougan, Solenzo, and Yako (Fig. 1), attending primary healthcare facilities between 2015 (pre-Gratuité intervention) and 2018 (post-intervention).

Data from the districts of Solenzo, Toma, and Yako included pre- and post-intervention records, enabling formal analysis. As of 2018, approximately 33% of the population in the Solenzo, Toma, and Yako health districts lived more than 4 km from the nearest health facility. All health centers in these districts are primary-level facilities, with no hospital infrastructure present [34]. Districts lacking baseline data (Dori, Dedougou, Ouahigouya, Nouna, Titao, and Tougan) were excluded from formal analyses but included in post-intervention trend analysis (Additional file 1).

A quasi-experimental difference-in-differences design was used to evaluate the free care policy’s effects on healthcare access and malaria outcomes among children under five years of age, leveraging variations in visit frequency, age, distance, and diagnosis. The evaluation used routine individual-level data from enrolled health districts, spanning one year prior to the intervention and two years post-intervention. Outcomes of interest included the average number of clinical visits per child per year and the annual prevalence of malaria and severe malaria among the under-five population. Children were stratified into two residence distance groups (< 5 km vs. ≥ 5 km from health facilities) to examine whether effects of the Gratuité policy differ by geographic accessibility.

### Variables

Formal analysis included several variables and covariates: the free care intervention period (baseline in 2015, implementation in 2016, and post-intervention from 2016–2018), distance to the visited health facility, child’s age, health district, and nutritional status (wasting and stunting). These variables were incorporated to capture temporal effects, geographic and demographic variation, and underlying health conditions that may influence outcomes.

Age was classified into five groups (1–5 years; Table 1). Since residence-to-health-facility distance metric data were unavailable, distances were computed using the shortest road network distance between the child’s village of residence and his/her visited health facility. We represented the road network as a graph $G=\left(V,E\right)$, where $V$ and $E$ denote the road’s nodes and edges weighted by geodetic distances ${w}_{ij}$. The weight ${w}_{ij}$ of each edge $\left(i,j\right)$ was computed using the Haversine formula: ${w}_{ij}=R\cdot \text{arccos}\left(\text{sin}\left({\phi }_{i}\right)\text{sin}\left({\phi }_{j}\right)+\text{cos}\left({\phi }_{i}\right)\text{cos}\left({\phi }_{j}\right)\text{cos}\left({\lambda }_{j}-{\lambda }_{i}\right)\right),$ where $R$ is the Earth’s radius, and $\left(\phi ,\lambda \right)$ are the latitude and longitude coordinates of the nodes. We then applied the algorithm $d\left( {x,y} \right) = \min \mathop \sum \limits_{{\left( {i,j} \right) \in P}} w_{ij}$ to identify the shortest path between the node representing village x and the node representing health facility y, minimizing the cumulative weights along the road. Continuous distances were categorized into < 5 km and ≥ 5 km to differentiate populations living within and beyond the government’s 5 km benchmark.

**Table 1:** Gratuité patients’ distribution by distance and various factors

Category	Factors	Distance to the nearest health facility			
		<5 km		≥ 5 km	
		N	%	N	%
Number of visits	Intervention				
	Pre-Gratuité	13,155	74	4,574	26
	Post-Gratuité	246,020	75	81,186	25
	Total	259,175	75	85,760	25
Number of patients	Intervention				
	Pre-Gratuité	11,406	74	4,041	26
	Post-Gratuité	133,050	72	51,167	28
	Age group				
	1year	42,956	72	17,023	28
	2 years	34,607	71	14,043	29
	3 years	28,510	72	10,977	28
	4 years	21,751	74	7,634	26
	5 years	16,632	75	5,531	25
	Malaria status				
	Mild malaria	153,678	75	52,276	25
	Severe malaria	5,343	72	2,107	28
	Nutritional status				
	Wasting	7,136	71	2,952	29
	Stunting	31,499	74	11,180	26
	Health district				
	Solenzo	88,803	73	33,526	27
	Toma	30,803	74	10,595	26
	Yako	24,850	69	11,087	31
	Total	144,456	72	55,208	28

### Data collection

Routine primary health data were collected from primary health facilities using the Electronic Medical Record (EMR) [31, 32], a tablet-based platform adopted by the government of Burkina Faso and introduced by Terre des Hommes progressively since 2010. The EMR platform is the WHO’s digital Integrated Management of Childhood Illness (IMCI) protocol [33]; it assists health workers in diagnosing and treating common childhood illnesses, ensuring adherence to clinical protocols. The platform records geocoded facility locations, clinical visit details, and patient residence. In Burkina Faso’s national health system, clinical visits and disease cases are typically first recorded at primary health facilities [21]. Even severe malaria cases are generally screened at this level before referral to higher-level hospitals, following national protocols. This ensures that primary health facilities data comprehensively capture service utilization and disease burden, with minimal risk of patients bypassing primary care.

### Causal inference analysis of the effects of the Gratuité policy

We used the canonical two-period Difference-in-Differences (DiD) framework, which compares one pre-policy time point with one post-policy time point. While this structure is widely used [35, 36], our study extends it by incorporating multiple post-intervention periods, allowing us to estimate not only the average policy effect but also how it evolves over time. As part of our pre-analysis, we confirmed baseline comparability in clinical visits and malaria outcomes between distance groups and examined trends following the intervention. Parallel trends in malaria outcomes across distance groups during the post-intervention period suggest that broader system-level trends remained stable, supporting the validity of the DiD parallel trends assumption. Generalized Mixed Effects Models (GLMMs) were used within the DiD framework to estimate both fixed and random effects while accounting for clustered and hierarchical data. We assessed the Gratuité policy’s Pre-and post-intervention effects on child clinic visits, malaria prevalence, and severe malaria, controlling for intervention year, distance to facility, age, district, and nutritional status. Secondary factors (gender, caregiver education, wealth, malaria prevention such as the use of ITN, SMC or IPTi) were unavailable.

### Gratuité policy effects on malaria

Malaria prevalence and severe malaria prevalence were modeled with a binomial GLMM as follow: $\text{log}\left(\frac{{p}_{it}}{1-{p}_{it}}\right)={\beta }_{0}+{\beta }_{1}{\text{Year}}_{t}+{\beta }_{2}{\text{Distance}}_{i}+{\beta }_{3}\left({\text{Year}}_{t}\times {\text{Distance}}_{i}\right)+{X}_{it}\gamma +{u}_{i}$

where: ${p}_{it}$ is the probability of the event (malaria, severe malaria) for individual $i$ at time $t$; ${\beta }_{0}:$ Intercept; ${\beta }_{1}:$ Effect of year; ${\beta }_{2}$: Effect of distance to health facility; ${\beta }_{3}$: Effect of the interaction term; ${\text{Year}}_{t}$ is a binary indicator for the post-intervention period; ${\text{Distance}}_{i}$ is a binary indicator for the Distance group; $\left({\text{Year}}_{t}\times {\text{Distance}}_{i}\right)$ is the interaction term capturing the DiD effect; ${X}_{it}$ represents control covariates including age, health district and nutritional status (wasting and stunting); ${u}_{i}$ is the random effect for individual $i$.

### Gratuité policy effects on clinical visits

The number of clinical visits was modeled with a negative binomial GLMM as follow: $\text{log}\left({\mu }_{it}\right)={\beta }_{0}+{\beta }_{1}{\text{Year}}_{t}+{\beta }_{2}{\text{Distance}}_{i}+{\beta }_{3}\left({\text{Year}}_{t}\times {\text{Distance}}_{i}\right)+{X}_{it}\gamma +{u}_{i}+{\epsilon }_{it}$

where: ${\mu }_{it}$ is the expected clinical visit’s count for individual $i$ at time $t$;${\beta }_{0},{\beta }_{1},{\beta }_{2},{\beta }_{3},and {b}_{j}$,${\text{Year}}_{t}$,${\text{Distance}}_{i}$,$\left({\text{Year}}_{t}\times {\text{Distance}}_{i}\right)$,${X}_{it}$, ${u}_{i}$ are as defined in the binomial model; and ${\epsilon }_{it}$ is the residual error term.

### Bayesian Hierarchical modeling and forecasting

Bayesian hierarchical models were used to predict malaria and severe malaria prevalence, as well as clinical visits for 2019–2020, based on baseline (2015) and intervention (2016–2018) data. To enhance interpretability for policy and forecasting, we applied Bayesian generalized linear models with a time-by-distance interaction. This structure captures divergent group trends and approximates nonlinearities, while supporting clear short-term extrapolation without post-hoc interpretation (e.g., spline disentangling). Posterior distributions

$$P\left(\theta |\mathbf{y}\right)=\frac{P\left(\mathbf{y}|\theta \right)\times P\left(\theta \right)}{P\left(\mathbf{y}\right)}$$

Bayes’ theorem using MCMC sampling, with convergence assessed through trace plots, effective sample sizes, and Gelman–Rubin statistics (R̂ < 1.01). Model selection relied on leave-one-out cross-validation (LOO-CV) to compare expected log pointwise predictive density (ELPD), with predictive checks ensuring replication of observed patterns.

### Forecasting malaria

Malaria or severe malaria prevalence $\left({y}_{ij}\right)$ for individual j in year i was modeled as a binomial outcome: ${y}_{ij}\sim {\text{Binomial}}\left({n}_{ij},{\theta }_{ij}\right)$ where: ${n}_{ij}$: Total number of malaria tests and ${\theta }_{ij}$: Probability of a positive malaria test. The logit-transformed probability ${\theta }_{ij}$ was expressed as:

${\text{logit}}\left({\theta }_{ij}\right)={\beta }_{0}+{\beta }_{1}({\text{Year}}_{i}\times {\text{Distance}}_{j})+{b}_{j}$ where ${b}_{j}\sim \mathcal{N}\left(0,{\sigma }_{b}^{2}\right)$: Random intercept for subject j.

### Forecasting clinical visits

Clinical visits $\left({y}_{ij}\right)$ for individual j in year i were modeled as a negative binomial outcome to account for overdispersion: ${y}_{ij}\sim {\text{NegativeBinomial}}\left({\mu }_{ij},\phi \right)$ where: ${\mu }_{ij}:$ Expected number of clinical visits and $\phi :$ Dispersion parameter.

The log-transformed expected count ${\mu }_{ij}$ was expressed as:

$$\text{log}\left({\mu }_{ij}\right)={\beta }_{0}+{\beta }_{1}({\text{Year}}_{i}\times {\text{Distance}}_{j})+{b}_{j}$$

New datasets were simulated for the prediction years (2019–2020), accounting for the covariates ${\text{Year}}_{i} and {\text{Distance}}_{j}.$ Random effects $\left({b}_{j}\right)$ were sampled from posterior distributions to reflect individual-level variation. Posterior predictions for clinical visits, malaria, and severe malaria prevalence were sampled from negative binomial or binomial distributions and summarized with means and 95% credible intervals. All three models showed strong convergence (Gelman-Rubin < 1.01, effective sample sizes > 1000), with trace plots and posterior densities confirming stable sampling (details in Additional file 2).

**Fig. 2:**
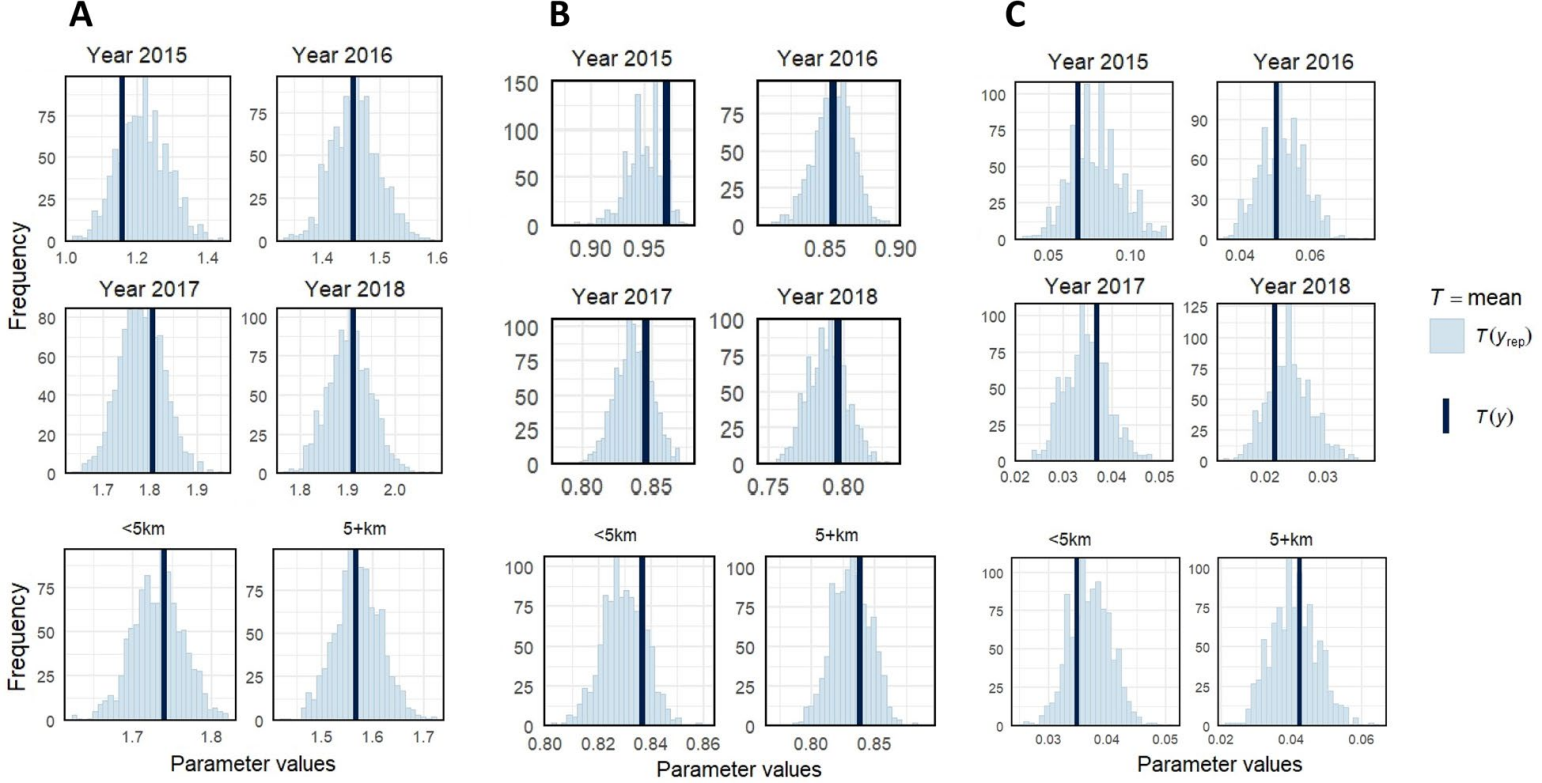
Posterior predictive checks by time and distance across models. **A:** Observed (T(y)) vs. Simulated (T(yrep)) mean number of Clinical Visits; **B:** Observed (T(y)) vs. Simulated (T(yrep)) mean of Malaria Prevalence; **C:** Observed (T(y)) vs. Simulated (T(yrep)) mean of Severe Malaria Prevalence. "NB: 5+km = ≥ 5 km"

**Fig. 3:**
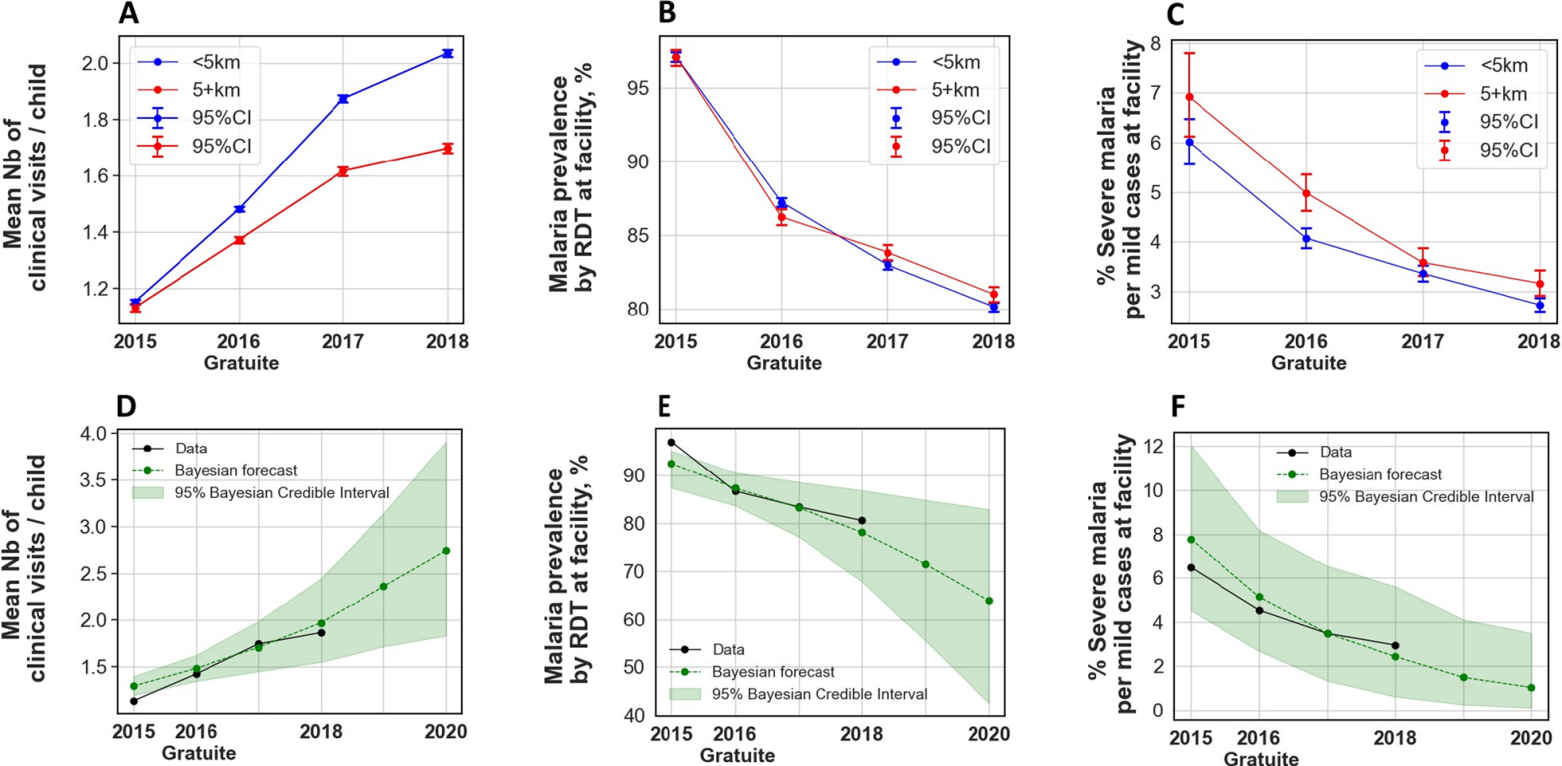
Clinical visits, malaria prevalence and case severity before and after the Gratuité policy intervention. "NB: 5+km = ≥ 5 km"

Posterior predictive checks across time (2015–2018) and distance groups (< 5 km, ≥ 5 km) showed observed statistics generally centered within simulated distributions, indicating consistent model fit. Occasional edge values did not suggest systematic misfit (Fig. 2). Overall, predictive checks, diagnostics, and alignment with observed trends (Fig. 3) support the models’ validity for policy forecasting.

To compare baseline differences between distance groups, we used the Mann–Whitney U test for clinical visits and a two-proportions test for malaria prevalence, given their respective non-normal and binary distributions. All statistical analyses were performed in R version 4.4.1. In all analyses, statistical significance was determined using a threshold of *p*-value < 0.05.

## Results

### Population demographics across distance, pre- and post- Gratuité policy

A total of 199,664 children from 192 villages visited 52 health facilities between 2015 and 2018 generating a total of 344,935 clinical visits associated with demographic, malariometric, nutrition and health outcome information per child (Table 1).

### Effects of the Gratuité policy and future forecasts

Data presented in Fig. 3 provide a comprehensive overview of the intervention’s effects on health service utilization and disease prevalence over time. Overall, at baseline (2015), clinical visits, malaria prevalence and severe malaria were on average similar between children living within 5 km of a health facility and those living farther away. There were no statistically significant differences between the two groups in clinical visits (W = 24,551,703, *p*-value = 0.2, Panel A), malaria prevalence (χ2 = 0.09, *p* = 0.6, Panel B) or severe malaria (χ2 = 3.1, *p* = 0.08, Panel C).

The panels collectively illustrate trends in clinical visits, malaria prevalence, and severe malaria prevalence, as well as forecasts based on Bayesian modeling. We limited the forecast to a two-year horizon 2019–2020 to ensure results remained empirically grounded and responsive to political instability, climate resilient, shifting policy and funding conditions. Our short-term forecasts offer a more cautious and actionable basis for planning and early evaluation. These visualizations highlight both observed and projected changes, offering insights into the temporal and spatial effects of the intervention on health outcomes for children in the study population. Figure 3-Panel A illustrates the mean number of clinical visits per child per year from 2015 (baseline) to 2018, comparing two groups based on proximity to health facilities: children living within 5 km (< 5 km) and those living beyond 5 km (≥ 5 km).

At baseline (2015), both groups exhibit a similar mean number of clinical visits (1.1). Following the intervention in 2016, the < 5 km group demonstrates higher utilization (1.5 visits) compared to the ≥ 5 km group (1.4 visits). This disparity widens over time, with the < 5 km group increasing to 1.9 and 2.2 visits in 2017 and 2018, respectively, while the ≥ 5 km group shows slower growth to 1.5 and 1.7 visits. Despite significant increases in clinical visits for both groups, the gap in utilization between them persists and significantly expands over time (*p* < 0.001, Table 2). Figure 3-Panel B shows a significant decrease in malaria prevalence across both groups, starting from 98% at baseline in 2015 to 85% in 2016, and further declining to 83% and 80% in 2017 and 2018, respectively. No notable differences are observed between the < 5 km and ≥ 5 km groups throughout the study period. Figure 3-Panel C highlights the prevalence of severe malaria, which follows a similar decreasing trend in both groups without significant differences. At baseline, prevalence rates are 6% and 7% for the < 5 km and ≥ 5 km groups, respectively. These rates decline to approximately 4.5% in 2016 and continue to fall to 3.1% and 2.5% in 2017 and 2018. We further found that nutritional status of wasting positively associated with severe malaria (*p* < 0.001, Table 2). Figure 3—Panel D combines observed data and Bayesian model projections for the mean number of clinical visits per child per year. Observed data show a significant increase from 1.2 clinical visits per child on average at baseline (2015) to 1.5 in 2016, 1.8 in 2017, and 2 clinical visits in 2018 (*p* < 0.001, Table 2). Bayesian modeling forecasts a continued upward trend, predicting a rise from 2.4 visits in 2019 to 2.7 visits in 2020. Figure 3-Panel E presents a similar visualization for malaria prevalence. Observed data show a significant decline from 98% at baseline to 80% in 2018 (*p* < 0.001, Table 2) with Bayesian modeling projecting further reductions from 71.5% in 2019 to 64% by 2020. Figure 3-Panel F depicts the trend in severe malaria prevalence, combining observed and forecasted data. Observed rates significantly decline from 6.5% to 3% in 2018 (*p* < 0.001, Table 2). Bayesian modeling predicts continued improvement, with rates falling from 1.5% in 2019 to 1% by 2020.

**Table 2:** Effect of Predictors on clinical visit and malaria outcomes pre-post Gratuité policy

Factor	Malaria prevalence			Severe malaria			Nb of visits per child		
	Estimate	Std error	p	Estimate	Std error	p	Estimate	Std error	p
Intercept	5.2524	0.3190	***	-2.8578	0.0511	***	0.2406	0.0581	***
2015 (Ref)									
2016 (Gratuité)	− 1.8939	0.0616	***	− 0.4529	0.0515	***	0.2563	0.0135	***
2017	− 2.2191	0.0609	***	− 0.6279	0.0508	***	0.4926	0.0132	***
2018	− 2.4026	0.0607	***	− 0.7904	0.0511	***	0.5742	0.0130	***
< 5km (Ref)									
≥ 5km	0.0426	0.1188		0.1350	0.0815		− 0.0186	0.0235	***
1 year old (Ref)									
2 years	− 0.8294	0.2983	***	0.0733	0.0362		− 0.0703	0.0572	
3 years	− 0.2141	0.2984	***	0.1664	0.0371		− 0.0960	0.0572	
4 years	− 0.0123	0.2987	***	0.0326	0.0422		− 0.120	0.0572	*
5 years	0.1113	0.2990	***	− 0.0808	0.0488		− 0.1253	0.0572	*
1 clinical visit (Ref)									
2 visits	− 0.3557	0.0997	***						
3 visits	− 0.4193	0.1030	***						
4 visits	− 0.3955	0.1102	***						
5 + visits	− 0.6660	0.1126	***						
Solenzo HD (ref)									
Toma	− 0.7439	0.0165	***	− 0.4866	0.0370	***	0.0353	0.0066	***
Yako	− 0.7894	0.0178	***	− 0.0593	0.0355		0.0023	0.0072	
2015: < 5km (Ref)									
2016:≥ 5km	− 0.0956	0.1225		− 0.0495	0.0958		− 0.0592	0.0261	*
2017:≥ 5km	0.0921	0.1216		− 0.0786	0.0956		− 0.1307	0.0257	***
2018:≥ 5km	0.1170	0.1211		0.0158	0.0961		− 0.1653	0.0255	***
No wasting (Ref)									
Wasting				0.5850	0.0461	***			
No Stunting (Ref)									
Stunting				0.0523	0.0305				

^.^ 0.05 < *p*-value < 0.09; *p-value < 0.05; **p-value < 0.01; ***p-value < 0.001; HD = Health District

### Changes in disease outcomes across geographies before and after Gratuité policy

**Fig. 4:**
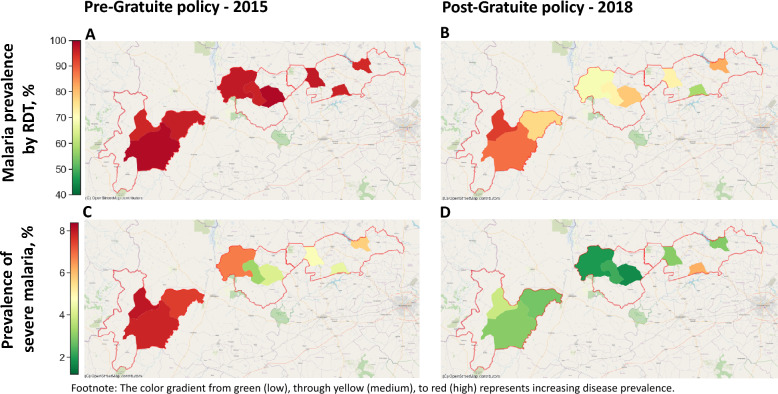
Geospatial distribution of malaria prevalence and case severity before and after the Gratuité policy intervention

In Fig. 4, we highlight the spatial distribution of malaria and severe malaria prevalence across the three intervention health districts before and after the intervention.

Using heat maps, the panels illustrate the baseline prevalence in 2015 and the substantial improvements observed by 2018, offering a detailed visual representation of the intervention’s effects at the communal (adm3) level. These maps provide crucial insights into the geographic variability and effectiveness of the intervention in reducing disease burden. Figure 4-Panel A presents a heat map of malaria prevalence in the three intervention health districts at baseline in 2015. The patches, corresponding to adm3 areas or communes, show uniformly high prevalence levels of 90–100%, as indicated by the intense red coloration, reflecting the critical malaria burden across all districts.

Figure 4-Panel B illustrates malaria prevalence post-intervention in 2018. The heat map reveals notable reductions, with District 4 (Solenzo) showing lower prevalence levels ranging from 75 to 90%, while Districts 2 (Toma) and 1 (Yako) experience the most significant declines, with prevalence levels ranging between 60 and 80% as shown by the map’s legend. The color gradient highlights the spatial heterogeneity of improvements across the intervention districts. Figure 4-Panel C depicts severe malaria prevalence at baseline in 2015. District 4 exhibits the highest prevalence, reaching up to 8%, while Districts 2 and 1 display moderately high prevalence levels ranging from 4 to 7%, as shown by the map’s legend. Figure 4-Panel D provides a post-intervention view of severe malaria prevalence in 2018, demonstrating substantial reductions across all districts. Prevalence levels drop to their lowest, around 2%, as represented by the lighter color tones in the heat map.

## Discussion

### Main findings

This study evaluates the effects of Burkina Faso’s Gratuité policy on healthcare utilization and malaria outcomes, focusing on children under five years of age. The findings revealed significant increases in healthcare access, as demonstrated by the rise in clinical visits. Concurrently, a marked decline in both malaria prevalence and severe malaria cases was observed, with additional insights derived from Bayesian modeling projections. These results align with studies showing that removing financial barriers through universal health policies significantly enhances healthcare-seeking behavior and reduces preventable disease burdens, particularly in vulnerable populations [37–39].

### Mechanisms of free healthcare’s effects

The removal of out-of-pocket payments is the primary pathway through which the free healthcare policy exerts its effects, substantially reducing financial barriers and encouraging greater healthcare-seeking behavior. The overall increase in clinical visits among children in our study is consistent with the policy’s primary objective of promoting service utilization. The post-intervention decline in malaria outcomes may reflect secondary benefits mediated by increased service utilization following the removal of financial barriers. The study also highlights persistent geographic disparities, as children living further from health facilities experienced slower improvements in healthcare service utilization compared to their closer counterparts suggesting that distance to health facilities negatively influences health seeking. This finding is consistent with evidence from other endemic settings, where rural and remote populations face barriers such as distance to health facilities, limited transportation, and low quality of care [5, 40–45]. In addition to the removal of user fees, socioeconomic factors such as education have been reported to influence health service utilization [39]; however, data on such confounders were not available for analysis in the present study.

### Broader context of malaria control

Malaria remains one of the leading public health challenges in sub-Saharan Africa, accounting for over 95% of global cases and deaths [46]. Despite significant investments in interventions such as insecticide-treated nets (ITNs) and seasonal malaria chemoprevention (SMC), progress has plateaued due to emerging challenges like insecticide and drug resistance [47, 48]. The findings from this study provide valuable evidence that health system strengthening initiatives like the Gratuité policy can complement malaria-specific interventions by increasing access to timely diagnosis and treatment, which are critical for reducing disease severity and mortality [45, 49–51]. While the model assessed the effects of the Gratuité policy on clinical visits and malaria outcomes, data on socioeconomic status and key preventive interventions, such as insecticide-treated nets (ITNs) and seasonal malaria chemoprevention (SMC), were not available for inclusion. These interventions are known to reduce malaria incidence and, if included, could have helped disentangle the contributions of clinical care versus prevention. Their absence limits the ability to fully attribute observed trends in malaria cases, particularly in regions or time periods where ITNs or SMC coverage may have varied.

### Geographic disparities and health system challenges

In our analyses, health district was statistically associated with both clinical visits and malaria, suggesting meaningful regional variation in policy outcomes. This highlights the importance of contextual factors in shaping the impact of the policy. Strengthening the free healthcare policy may require tailored strategies that address these underlying regional disparities. Such disparities highlight systemic barriers, including health system weaknesses like distance to health facilities [5], supply chain inefficiencies and provider shortages [52–56] as well as the effects of security challenges on health service access [57, 58]. While health district was included in our model to adjust for regional heterogeneity, the absence of data from the National Health Information System (NHIS) on structural determinants such as facility capacity, healthcare staffing, quality of care, or transport infrastructure limits deeper interpretation [59]. Addressing these disparities requires multisectoral approaches, such as investments in transportation infrastructure, strengthening community health worker (CHW) programs and their activities; and leveraging digital health solutions to improve equity and quality in care delivery and monitoring [16, 17, 20, 60].

### Policy and research implications

The Gratuité policy demonstrates the transformative potential of financial reforms to improve health outcomes. However, complementary strategies are needed to address inequities. Strengthening the CHW program could help bridge gaps in rural and semi-urban areas [17, 20, 61], while improved diagnostic tools, such as the EMR platform enhance the accuracy of malaria detection [32]. Furthermore, integrating malaria-specific interventions with broader health system reforms could help sustain progress [62–65]. Future research should evaluate the long-term sustainability of the Gratuité policy, particularly considering financial constraints and competing health priorities. Emerging technologies, such as malaria vaccines, offer additional avenues for exploration. Given that over 80% of Burkina Faso’s population resides in rural and semi-urban areas, the study findings from rural and semi-urban health districts could be generalizable to the majority of the country. The observed trends emphasize the importance of interventions targeting rural and semi-urban populations, where health inequities are often most pronounced. However, the generalizability to urban areas, which represent approximately 20% of the population, may be limited due to socioeconomic and infrastructural differences. For example, urban areas often have better access to health facilities, which could result in differential policy effects.

### Study limitations

The primary limitation of this study is the restricted geographic scope, as data were formally analyzed from only three of the country’s 70 health districts. Although these districts are representative of rural and semi-urban Burkina Faso, the exclusion of urban populations limits the ability to evaluate the policy’s broader effects across varying socioeconomic settings. Urban areas may face unique challenges, such as higher patient-to-provider ratios and the potential for overcrowding in health facilities, which could alter the effectiveness of the policy. Moreover, differences in district-specific factors such as population density, malaria transmission dynamics, and health system capacity may influence the observed outcomes. While the study provides robust insights for rural and semi-urban settings, additional research is needed to confirm these findings in urban settings, particularly those with distinct epidemiological or health system characteristics. Another limitation is the reliance on health facility-based data, which may underestimate malaria cases not captured through formal healthcare channels. Rural populations often rely on traditional healers or self-medication due to geographic and cultural barriers [66, 67], potentially biasing the results toward facility-attending populations. Future studies incorporating community-based surveys could address this gap. Despite these limitations, the findings contribute valuable evidence to the discourse on malaria control in sub-Saharan Africa.

## Conclusions

Our study highlights the potential of universal health policies like the Gratuité policy in increasing clinical visits and reducing malaria prevalence and severe malaria. However, achieving equitable health outcomes will require addressing persistent geographic and systemic inequities. These findings underscore the need for integrated, context-specific strategies that combine financial, technical, and operational innovations to increase service utilization. Such approaches are also vital to cope with malaria and strengthen health systems in Burkina Faso and across sub-Saharan Africa.

## Supplementary Information

Additional file1 (DOCX 131 KB)

Additional file2 (DOCX 401 KB)

## Data Availability

The data analyzed in this study is subject to the following licenses/restrictions: The data supporting this study's findings are available from health facilities patients’ registers. Data sharing does not apply to this article as it contains information that could compromise the privacy of research participants. Requests to access these datasets should be directed to Pierre Yameogo at yampite@gmail.com.
