# Supplementary material for: Evaluating the effects of the free healthcare policy on clinical visits and malaria among children in Burkina Faso: a modeling study of past trends and future forecasts
Source: Glob Health Res Policy. 2025 Dec 15;10:64. doi: 10.1186/s41256-025-00455-5 (PMC12703899; doi:10.1186/s41256-025-00455-5)
Supplement: Supplementary file 1 — Additional file1 (DOCX 131 KB) [file 41256_2025_455_MOESM1_ESM.docx]

**Additional file 1**

**Clinical visits, malaria prevalence and case severity from districts without baseline data.**

**
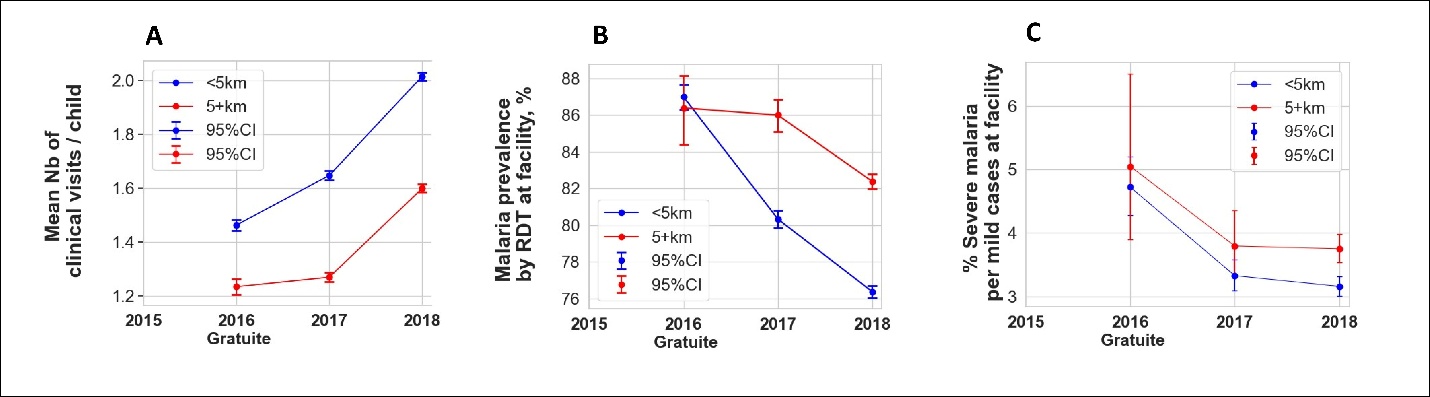
**
