# Supplementary material for: Evaluating the effects of the free healthcare policy on clinical visits and malaria among children in Burkina Faso: a modeling study of past trends and future forecasts
Source: Glob Health Res Policy. 2025 Dec 15;10:64. doi: 10.1186/s41256-025-00455-5 (PMC12703899; doi:10.1186/s41256-025-00455-5)

**Additional file 2**

1. **Trace plots and posterior densities for Clinical Visit model parameters**

**
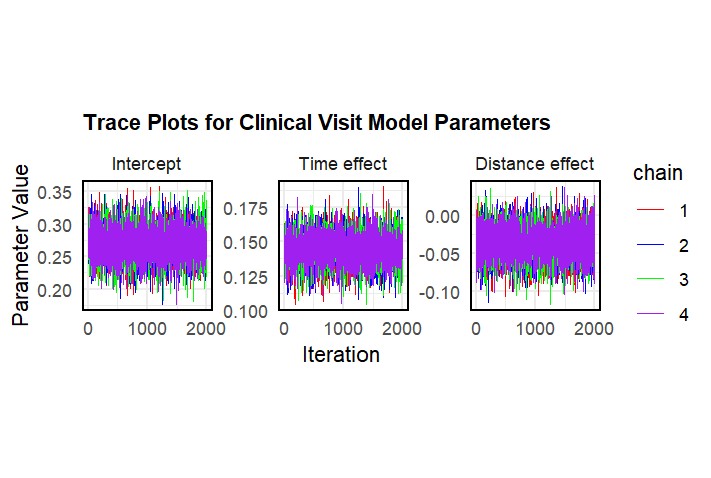
**

**
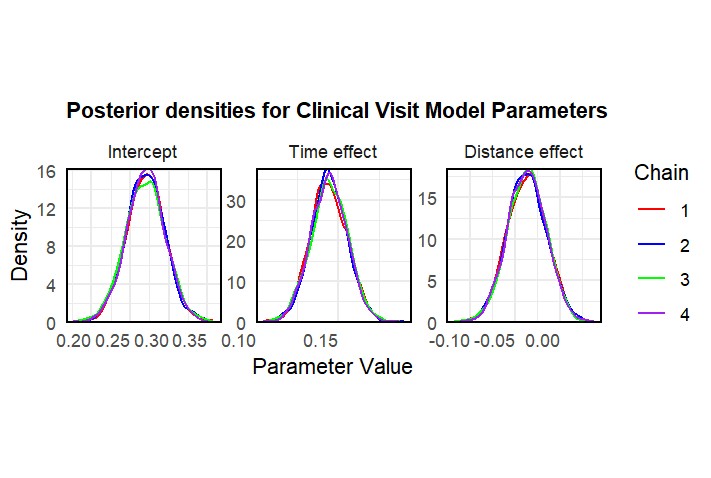
**

1. **Trace plots and posterior densities for Malaria Prevalence model parameters**


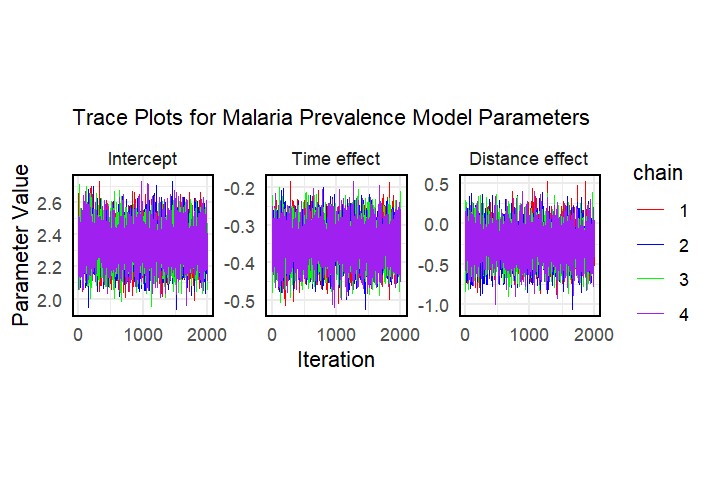


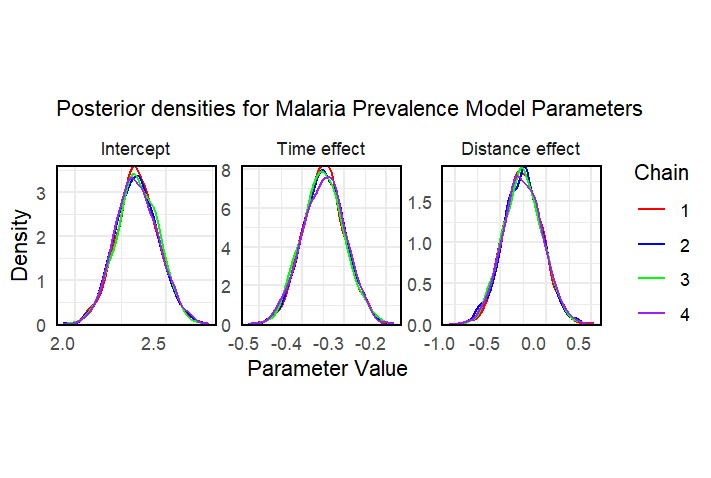


1. **Trace plots and posterior densities for Severe Malaria model parameters**


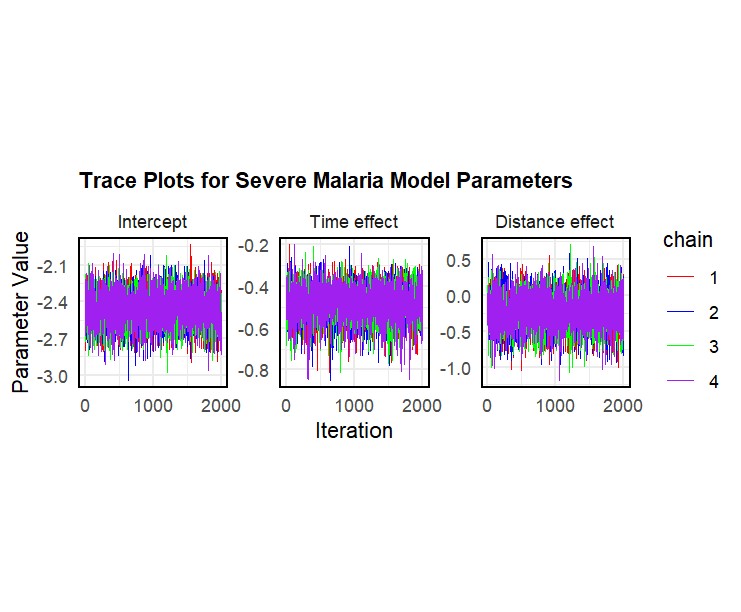


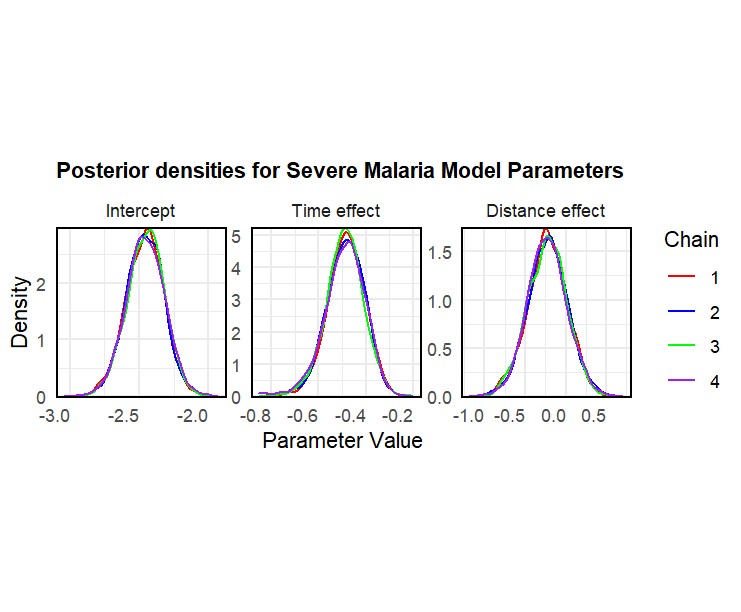

Supplement: Supplementary file 2 — Additional file2 (DOCX 401 KB) [file 41256_2025_455_MOESM2_ESM.docx]
